# Bridging the Artificial Intelligence (AI) Divide: Do Postgraduate Medical Students Outshine Undergraduate Medical Students in AI Readiness?

**DOI:** 10.7759/cureus.67288

**Published:** 2024-08-20

**Authors:** Rohankumar Gandhi, Alpesh Parmar, Jimmy Kagathara, Dhruv Lakkad, Jay Kakadiya, Yogesh Murugan

**Affiliations:** 1 Community and Family Medicine, Shri M. P. Shah Government Medical College, Jamnagar, IND; 2 Public Health, Shri M. P. Shah Government Medical College, Jamnagar, IND; 3 Community Medicine, Smt. B. K. Shah Medical Institute & Research Centre, Vadodara, IND; 4 Internal Medicine, Shri M. P. Shah Government Medical College, Jamnagar, IND; 5 Family Medicine, Guru Gobind Singh Government Hospital, Jamnagar, IND

**Keywords:** healthcare technology, postgraduate medical students, undergraduate medical students, medical education, artificial intelligence

## Abstract

Introduction: As artificial intelligence (AI) transforms healthcare, medical education must adapt to equip future physicians with the necessary competencies. However, little is known about the differences in AI knowledge, attitudes, and practices between undergraduate and postgraduate medical students. This study aims to assess and compare AI knowledge, attitudes, and practices among undergraduate and postgraduate medical students, and to explore the associated factors and qualitative themes.

Methods: A mixed-methods study was conducted, involving 605 medical students (404 undergraduates, 201 postgraduates) from a tertiary care center. Participants completed a survey assessing AI knowledge, attitudes, and practices. Semi-structured interviews and focus group discussions were conducted to explore qualitative themes. Quantitative data were analyzed using descriptive statistics, t-tests, chi-square tests, and regression analyses. Qualitative data underwent thematic analysis.

Results: Postgraduate students demonstrated significantly higher AI knowledge scores than undergraduates (38.9±4.9 vs. 29.6±6.8, p<0.001). Both groups held positive attitudes, but postgraduates showed greater confidence in AI's potential (p<0.001). Postgraduates reported more extensive AI-related practices (p<0.001). Key qualitative themes included excitement about AI's potential, concerns about job security, and the need for AI education. AI knowledge, attitudes, and practices were positively correlated (p<0.01).

Conclusions: This study reveals a significant AI knowledge gap between undergraduate and postgraduate medical students, highlighting the need for targeted AI education. The findings can inform curriculum development and policies to prepare medical students for the AI-driven future of healthcare. Further research should explore the long-term impact of AI education on clinical practice.

## Introduction

Artificial Intelligence (AI) is rapidly transforming the landscape of healthcare, with the potential to revolutionize medical diagnosis, treatment, and decision-making [1]. AI technologies, such as machine learning and natural language processing, have demonstrated remarkable capabilities in analyzing complex medical data, identifying patterns, and providing personalized recommendations [2]. As AI continues to advance, medical professionals must possess the knowledge, skills, and attitudes necessary to effectively integrate these technologies into their practice [3].

Medical education plays a pivotal role in preparing future physicians for the AI-driven healthcare environment [4]. However, current medical curricula often lag in equipping students with the requisite AI competencies [5]. Studies have reported limited AI knowledge and varying attitudes toward AI among medical students [6,7]. This knowledge gap and attitudinal variability can hinder the effective adoption and utilization of AI tools in clinical settings [8].

Moreover, there is a paucity of research examining the differences in AI knowledge, attitudes, and practices between undergraduate and postgraduate medical students [9]. Understanding these differences is crucial for designing targeted educational interventions and ensuring a smooth transition from medical school to residency and beyond [10]. Postgraduate students, being at a more advanced stage of their training and often exposed to clinical applications of AI, may possess different levels of knowledge and perspectives compared to their undergraduate counterparts [11].

Therefore, this study aimed to bridge the gap in the literature by conducting a comprehensive assessment of AI knowledge, attitudes, and practices among undergraduate and postgraduate medical students. The specific objectives were the following: to assess and compare the level of AI knowledge between undergraduate and postgraduate medical students across various domains, including basic concepts, medical applications, ethical considerations, and clinical reasoning; to examine and contrast the attitudes toward AI in medicine held by undergraduate and postgraduate students, including perceived benefits, concerns, and future expectations; to investigate and compare the AI-related practices and experiences of undergraduate and postgraduate students, such as exposure to AI tools, research participation, and planned integration of AI in future practice; to explore the qualitative themes underlying students' perspectives on AI in medicine, providing a deeper understanding of their knowledge, attitudes, and practices; and to identify the factors associated with higher levels of AI knowledge, positive attitudes, and engagement with AI practices among medical students.

By addressing these objectives, this study contributes to the growing body of literature on AI in medical education and provides valuable insights for curriculum development, policy-making, and future research. The findings can inform the design of educational interventions tailored to the specific needs of undergraduate and postgraduate students, ultimately promoting the effective integration of AI in medical practice and improving patient care in the era of AI.

## Materials and methods

Study design and setting

This study employed a mixed-methods approach to comprehensively assess the knowledge, attitudes, and practices regarding AI in medicine among undergraduate and postgraduate medical students. The study was conducted at a tertiary care center following approval from the Institutional Review Board (Shri M. P. Shah Medical College and Guru Gobind Government Hospital, Ref. No. 01/01/2023) [12,13].

Sample size

The target sample size for this study was determined using a priori power analysis conducted with G*Power software (Heinrich Heine University Düsseldorf, Düsseldorf, Germany) [14]. The power analysis was based on the following parameters: significance level (α) = 0.05, power (1-β) = 0.80, and effect size (Cohen's d) = 0.5 (medium effect).

The effect size was chosen based on previous studies examining knowledge and attitudes toward health technology among medical students [15,16]. A medium effect size was a conservative estimate that balanced the likelihood of detecting a meaningful difference with the feasibility of recruitment. The power analysis was conducted for the primary comparison of interest, which was the difference in AI knowledge scores between undergraduate and postgraduate students. The required sample size was calculated for an independent samples t-test. To account for potential non-response and incomplete data, the target sample size was inflated by 20%.

For the qualitative component of the study, we employed purposive sampling to ensure a diverse representation of perspectives from both undergraduate and postgraduate students. The sample size for the qualitative phase was determined using the principle of data saturation [13]. We initially conducted 20 semi-structured interviews (10 undergraduate and 10 postgraduate students) and four focus group discussions (two for each group, with six to eight participants per group). Data collection continued until thematic saturation was reached, which occurred after an additional five interviews and one focus group discussion.

Sampling technique

A two-stage stratified random sampling technique was employed to ensure adequate representation across key subgroups (undergraduate vs. postgraduate, year of study). This approach minimized selection bias and enhanced the generalizability of findings [17-19]. The sampling frame included all medical students enrolled in the medical college and hospital where this study was conducted. This single-center approach was chosen due to feasibility constraints and to ensure consistency in the educational environment of the participants.

In the first stage, the sampling frame was stratified by education level (undergraduate vs. postgraduate). Within each stratum, participants were randomly selected using a computer-generated random number sequence. This ensured that the proportion of undergraduate and postgraduate students in the sample reflected their distribution in the overall population. In the second stage, the selected participants were further stratified by year of study (e.g., first year, second year, etc.). Within each year-of-study stratum, participants were again randomly selected using a computer-generated random number sequence. This ensured that each year of study was adequately represented within the undergraduate and postgraduate subsamples. The number of participants selected from each stratum was proportional to the stratum's size in the overall population. This approach yielded a sample representative of the target population in terms of education level and year of study [12].

Eligibility criteria

The study included both undergraduate and postgraduate medical students. The inclusion criteria were: (1) currently enrolled in an accredited medical program (Bachelor of Medicine, Bachelor of Surgery (MBBS) for undergraduates or Doctor of Medicine/Master of Surgery (MD/MS) for postgraduates) at the medical college and its affiliated hospital at the time of data collection, (2) aged 18 years or older, and (3) able to provide informed consent. Students who were on extended leave or studying abroad during the data collection period were excluded. Visiting students from other institutions were excluded. Students enrolled in super-specialty courses (Doctorate of Medicine/Master of Chirurgiae (DM/MCH)) were also excluded, as their experiences might differ significantly from the general postgraduate population.

Data collection methods

Data were collected through an online survey platform. Eligible participants received an email invitation containing a unique link to the survey. The survey consisted of four sections: (1) demographic information, (2) AI knowledge assessment, (3) attitudes toward AI in medicine, and (4) AI technology acceptance and usage. The knowledge assessment was developed based on a review of AI competencies for healthcare professionals [8] and pilot-tested with a sample of (n=20) medical students to ensure clarity and content validity. The attitudes and technology acceptance sections were adapted from validated instruments [20-25]. Participants could complete the survey at their convenience within a two-week window. Weekly reminder emails were sent to non-responders to maximize the response rate. Data from incomplete surveys were included in the analysis if at least 80% of the items were answered (see Appendices).

Qualitative component

The semi-structured interviews were conducted using an interview guide developed based on the literature review and preliminary quantitative findings. The guide covered topics such as personal experiences with AI in medical education, perceived benefits and challenges of AI integration, and suggestions for improving AI education. Interviews lasted approximately 45-60 minutes and were audio-recorded with participants' consent. Focus group discussions were facilitated by trained moderators using a discussion guide that encouraged interactive dialogue among participants. These sessions, lasting 90-120 minutes, explored shared experiences, group dynamics, and collective perspectives on AI in medicine. All discussions were video recorded to capture both verbal and non-verbal communication.

Data analysis

Data analysis involved both quantitative and qualitative techniques. Quantitative data were analyzed using IBM SPSS Statistics for Windows, Version 23, (Released 2015; IBM Corp., Armonk, New York, United States). We used descriptive statistics to summarize participant characteristics and survey responses. Independent samples t-tests and chi-square tests were used to compare continuous and categorical variables, respectively, between undergraduate and postgraduate students. Normality assumptions were assessed using Shapiro-Wilk tests and visual inspection of Q-Q plots and histograms. Non-parametric alternatives (Mann-Whitney U test) were used for non-normally distributed data. Effect sizes were calculated using Cohen's d for continuous variables (0.2: small, 0.5: medium, and 0.8: large) and Cramér's V for categorical variables (0.1: small, 0.3: medium, and 0.5: large) [19].

We employed multivariable linear regression to identify predictors of AI knowledge scores. Multivariable linear regression was used to identify predictors of AI knowledge scores. The regression model included education level, year of study, gender, and prior AI experience as independent variables. Model assumptions (linearity, homoscedasticity, independence, normality) were assessed by examining standardized residual plots and variance inflation factors (VIFs). All tests were two-sided and p-values less than 0.05 were considered statistically significant. Missing data were handled using pairwise deletion. For qualitative data, we conducted a thematic analysis of interview and focus group transcripts using NVivo software (Lumivero, Denver, Colorado, United States), applying the constant comparative method to identify emerging themes [20,22] (Table 1).

**Table 1:** Operational definitions and validation process The operational definitions were adapted from [1,23].

Concept	Operational definition	Validation process
Artificial intelligence (AI) in medicine	The use of machine learning algorithms and other cognitive technologies in diagnosis, treatment planning, patient monitoring, and healthcare administration	Content validity: Reviewed by a panel of five experts in medical education, AI in healthcare, and survey methodology
AI knowledge	Understanding of basic AI concepts, applications in medicine, limitations, and ethical considerations, as measured by the knowledge assessment questionnaire	Face validity: Piloted with 20 medical students (10 undergraduate (UG), 10 postgraduate (PG)) for clarity, readability, and appropriateness of language
Attitudes toward AI	Beliefs, feelings, and behavioral intentions regarding the use of AI in medical practice, as measured by the attitude assessment scale	Construct validity: Exploratory factor analysis (EFA) conducted on data from a pilot sample of 150 students
AI tool usage	Practical experience with AI-based technologies in medical education or clinical settings, as reported in the practice assessment questionnaire	Criterion validity: AI knowledge scores correlated with performance on a separate AI competency test (r = 0.72, p < 0.001)
Clinical reasoning	The cognitive process used by healthcare providers to investigate, evaluate, and determine the best course of action for patient care	Internal consistency reliability: Cronbach's alpha calculated for each subscale (range: 0.78 to 0.89)
		Test-retest reliability: Intraclass correlation coefficients (ICCs) ranged from 0.81 to 0.92 in a subset of 50 participants who completed the questionnaire twice, two weeks apart

## Results

Demographic data (Table 2 ) revealed significant age differences between the two groups, with undergraduates having a mean age of 22.3 ± 2.1 years compared to 28.7 ± 3.5 years for postgraduates (p<0.001). Gender distribution was relatively balanced, with a slight female majority among undergraduates (218/404, 54%) and a slight male majority among postgraduates (109/201, 54%), though this difference was not statistically significant (p=0.137). The year of study distribution was fairly even among undergraduates, while postgraduates were more concentrated in the second (70/201, 34.7%) and third years (71/201, 35.3%) of their programs.

**Table 2:** Demographic characteristics of study participants (N=605) Data are presented as mean ± standard deviation (SD) for age and N (%) for gender and year of study. **p < 0.01: highly significant

Characteristic	Undergraduates (n=404)	Postgraduates (n=201)	p-value
Age (years)			
Mean ± SD	22.3 ± 2.1	28.7 ± 3.5	<0.001 **
Gender			
Male	186 (46%)	109 (54%)	0.137
Female	218 (54%)	92 (46%)	
Year of study			
First year	101 (25%)	60 (30%)	0.286
Second year	111 (27.5%)	70 (34.7%)	
Third year	97 (24%)	71 (35.3%)	
Fourth year	95 (23.5%)	N/A	

Knowledge assessment results (Table 3) consistently showed that postgraduate students scored higher than undergraduate students across all areas of AI knowledge. The overall knowledge score (out of 50) was significantly higher for postgraduates (38.9 ± 4.9) compared to undergraduates (29.6 ± 6.8) (p<0.001). Postgraduates demonstrated a greater understanding of basic AI concepts (7.8 ± 1.5 vs. 6.2 ± 1.8), AI applications in medicine (8.1 ± 1.3 vs. 5.7 ± 2.0), limitations of AI in healthcare (7.5 ± 1.6 vs. 5.9 ± 1.7), ethical considerations (7.9 ± 1.2 vs. 6.5 ± 1.5), and AI in clinical reasoning (7.6 ± 1.4 vs. 5.3 ± 1.9). All these differences were statistically significant (p<0.001), indicating a substantial knowledge gap between the two groups.

**Table 3:** Knowledge assessment of the participants (N=605) Scores are on a scale of 0-10 for individual areas and 0-50 for overall knowledge. **p < 0.01: highly significant

Knowledge area	Undergraduates (n=404)	Postgraduates (n=201)	p-value
Basic AI concepts (mean score ± SD)	6.2 ± 1.8	7.8 ± 1.5	<0.001 **
AI applications in medicine (mean ± SD)	5.7 ± 2.0	8.1 ± 1.3	<0.001 **
Limitations of AI in healthcare (mean ± SD)	5.9 ± 1.7	7.5 ± 1.6	<0.001 **
Ethical considerations (mean ± SD)	6.5 ± 1.5	7.9 ± 1.2	<0.001 **
AI in clinical reasoning (mean ± SD)	5.3 ± 1.9	7.6 ± 1.4	<0.001 **
Overall knowledge score (mean ± SD)	29.6 ± 6.8	38.9 ± 4.9	<0.001 **

Attitude assessment (Table 4), measured on a 5-point Likert scale, revealed that both groups generally held positive attitudes toward AI in medicine, with postgraduates showing more favorable attitudes across all statements. Postgraduates were significantly more likely to believe that AI will improve patient care (4.5 ± 0.6 vs. 4.2 ± 0.8, p=0.001) and that AI is essential for future medical practice (4.6 ± 0.5 vs. 4.1 ± 0.9, p<0.001). Interestingly, undergraduates were more concerned about AI replacing human doctors (2.8 ± 1.2 vs. 2.3 ± 1.1, p<0.001) and leading to a loss of human touch in medicine (3.5 ± 1.1 vs. 3.2 ± 1.2, p=0.015). Regarding clinical reasoning, postgraduates showed more positive attitudes toward AI's ability to enhance clinical reasoning skills (4.2 ± 0.7 vs. 3.8 ± 0.9, p<0.01) and the usefulness of AI tools in clinical reasoning education (4.3 ± 0.6 vs. 3.9 ± 0.8, p<0.001).

**Table 4:** Attitude assessment of the participants (N=605) Data are presented as mean ± SD. Scores are on a 5-point Likert scale (1 = strongly disagree, 5 = strongly agree). *p < 0.05: significant, **p < 0.01: highly significant

Attitude statement	Undergraduates (n=404)	Postgraduates (n=201)	p-value
AI will improve patient care	4.2 ± 0.8	4.5 ± 0.6	0.001 **
AI may replace human doctors	2.8 ± 1.2	2.3 ± 1.1	0.001 **
AI is essential for future medical practice	4.1 ± 0.9	4.6 ± 0.5	0.001 **
I feel comfortable using AI in my future practice	3.7 ± 1.0	4.2 ± 0.7	0.001 **
AI may lead to the loss of human touch in medicine	3.5 ± 1.1	3.2 ± 1.2	0.015 *
AI can enhance clinical reasoning skills	3.8 ± 0.9	4.2 ± 0.7	0.001 **
AI tools are useful in clinical reasoning education	3.9 ± 0.8	4.3 ± 0.6	0.001 **

Practice assessment (Table 5) highlighted significant differences between the two groups in their engagement with AI in medicine. Postgraduates were more likely to have used AI tools in their medical studies (141/201, 70% vs. 137/404, 34%, p<0.001), attended AI in Medicine workshops/seminars (121/201, 60% vs. 91/404, 22.5%, p<0.001), read scientific papers on AI in medicine (161/201, 80% vs. 166/404, 41%, p<0.001), and participated in AI-related research (60/201, 30% vs. 30/404, 7.5%, p<0.001). A higher percentage of postgraduates also planned to integrate AI into their future practice (181/201, 90% vs. 263/404, 65%, p<0.001). Notably, postgraduates had significantly more exposure to AI-enhanced clinical reasoning tools (90/201, 45% vs. 61/404, 15%, p<0.001) and were more likely to have used AI-based clinical decision support systems (70/201, 35% vs. 40/404, 10%, p<0.001).

**Table 5:** Practice assessment of the participants (N=605) Data are presented as N (%). **p < 0.01: highly significant

Practice item	Undergraduates (n=404)	Postgraduates (n=201)	p-value
Used AI tools in medical studies	137 (34%)	141 (70%)	<0.001 **
Attended AI in Medicine workshop/seminar	91 (22.5%)	121 (60%)	<0.001 **
Read scientific papers on AI in medicine	166 (41%)	161 (80%)	<0.001 **
Participated in AI-related research	30 (7.5%)	60 (30%)	<0.001 **
Plan to integrate AI in future practice	263 (65%)	181 (90%)	<0.001 **
Exposed to AI-enhanced clinical reasoning tools	61 (15%)	90 (45%)	<0.001 **
Used AI-based clinical decision support systems	40 (10%)	70 (35%)	<0.001 **

Correlation analysis (Table 6) revealed significant positive associations between knowledge scores, positive attitudes toward AI, and AI tool usage. Age and education level (being postgraduate) were also positively correlated with these variables. Gender showed a weak positive correlation with knowledge scores and AI tool usage, but not with attitudes. The strongest correlations were observed between knowledge scores and positive attitudes (r=0.65), and between education level and AI tool usage (r=0.62).

**Table 6:** Associations between knowledge, attitudes, practices, and demographics Values represent Pearson correlation coefficients (r). *p < 0.05: significant, **p < 0.01: highly significant. Knowledge score: overall knowledge score from Table 2. Positive attitude: average score of positive attitude statements from Table 3. AI tool usage: binary variable (1 = has used AI tools in medical studies, 0 = has not used). Education level: binary variable (1 = postgraduate, 0 = undergraduate).

Variable	Knowledge score	Positive attitude	AI tool usage	Age	Gender (male)
Knowledge score	1.00	0.65**	0.58**	0.42**	0.15*
Positive attitude	0.65**	1.00	0.51**	0.38**	0.09
AI tool usage	0.58**	0.51**	1.00	0.45**	0.22**
Age	0.42**	0.38**	0.45**	1.00	0.05
Gender (male)	0.15*	0.09	0.22**	0.05	1.00
Education level (postgraduate)	0.53**	0.47**	0.62**	0.76**	0.11

Table 7 presents the qualitative themes that emerged from the study of AI in medicine among medical students. These themes provide deeper insights into the perceptions, concerns, and expectations of both undergraduate and postgraduate medical students regarding AI in healthcare. Below is a detailed explanation of each theme, supported by participants' phrases.

**Table 7:** Qualitative themes on artificial intelligence (AI) in medicine among medical students Frequency: very high (>75% of respondents), high (50-75%), moderate (25-49%), low (<25%) UG: undergraduate student; PG: postgraduate student

Theme	Description	Representative quotes	Frequency
Excitement about AI's potential	Students expressed enthusiasm about AI's capacity to improve diagnostics, treatment planning, and overall patient care	"AI could revolutionize how we detect diseases early" (UG). "I'm excited about AI's potential to assist in complex treatment decisions" (PG)	High
Concerns about job security	Worries about AI potentially replacing human doctors or reducing job opportunities	"I fear AI might make some medical specialties obsolete" (UG). "Will there be fewer residency spots if AI can do some of our jobs?" (PG)	Moderate
Ethical considerations	Reflections on privacy, data security, and the potential for bias in AI systems	"We need to ensure AI doesn't perpetuate existing health disparities" (PG). "Patient privacy with all this data is a big concern for me" (UG)	High
Need for AI education	A desire for more comprehensive AI training in the medical curriculum	"I wish we had a dedicated course on AI in medicine" (UG). "We need practical training on how to use AI tools in clinical settings" (PG)	Very high
The human touch in medicine	Emphasis on the irreplaceable aspects of human doctors, such as empathy and intuition	"AI can't replace the empathy and emotional support we provide" (UG). "There's an art to medicine that I don't think AI can fully replicate" (PG)	High
Integration challenges	Concerns about the practical aspects of incorporating AI into existing healthcare systems	"How will AI tools integrate with our current EMR systems?" (PG). "I worry about the learning curve for older physicians in adopting AI" (UG)	Moderate
Enhanced pattern recognition	Students reported AI tools helping them identify patterns in patient data more quickly	"The AI system pointed out correlations in lab results that I might have missed initially" (PG)	High
Improved diagnostic accuracy	Many students felt AI-enhanced tools improved their diagnostic skills	"Using the AI-based differential diagnosis tool made me consider conditions I hadn't thought of" (UG)	High
Concerns about over-reliance	Some students worried about becoming too dependent on AI tools	"I fear we might lose our ability to reason independently if we rely too much on AI suggestions" (UG)	Moderate
Integration challenges	Students noted difficulties in incorporating AI tools into existing clinical reasoning frameworks	"It's not always clear how to balance the AI's suggestions with what we've been taught traditionally" (PG)	Moderate

Excitement about AI’s potential (high frequency)

This theme reflected the enthusiasm and optimism students feel about AI's role in advancing medical practice. Many students saw AI as a powerful tool that could revolutionize various aspects of healthcare. An undergraduate student expressed: "AI could revolutionize how we detect diseases early." This statement highlighted the potential of AI in improving diagnostic processes, possibly leading to earlier interventions and better patient outcomes. A postgraduate student noted: "I'm excited about AI's potential to assist in complex treatment decisions." This comment suggested that more advanced students recognized AI's capability to support clinical decision-making, particularly in challenging cases.

Concerns about job security (moderate frequency)

While students were excited about AI, there was also apprehension about how it might affect their future careers. This theme was moderately prevalent, indicating it's a significant concern but not overwhelming. An undergraduate voiced their worry: "I fear AI might make some medical specialties obsolete." This reflected concerns about AI potentially replacing certain roles or specializations within medicine. A postgraduate student questioned: "Will there be fewer residency spots if AI can do some of our jobs?" This highlighted concerns about reduced job opportunities, even at the early career stage.

Ethical considerations (high frequency)

Students demonstrated a high level of awareness regarding the ethical implications of AI in healthcare. This theme encompassed issues of privacy, data security, and potential biases in AI systems. A postgraduate student emphasized: "We need to ensure AI doesn't perpetuate existing health disparities." This showed an understanding of how AI could potentially exacerbate inequalities in healthcare if not carefully implemented. An undergraduate expressed concern: "Patient privacy with all this data is a big concern for me." This highlighted awareness of the data privacy challenges that came with the increased use of AI in healthcare.

Need for AI education (very high frequency)

This was the most prevalent theme, indicating a strong desire among students for more comprehensive AI training in their medical education. An undergraduate student expressed: "I wish we had a dedicated course on AI in medicine." This suggested that current medical curricula might not be adequately addressing AI topics. A postgraduate student emphasized the need for practical skills: "We need practical training on how to use AI tools in clinical settings." This indicated that even at more advanced levels, there was a perceived lack of hands-on training with AI technologies.

The human touch in medicine (high frequency)

Despite enthusiasm for AI, students strongly emphasized the irreplaceable aspects of human doctors. This theme highlighted the perceived limitations of AI in medicine. An undergraduate stated: "AI can't replace the empathy and emotional support we provide." This underscored the belief that emotional intelligence and interpersonal skills remain crucial in patient care. A postgraduate reflected: "There's an art to medicine that I don't think AI can fully replicate." This suggested a recognition of the intuitive and experience-based aspects of medical practice that may be challenging to replicate with AI.

Integration challenges (moderate frequency)

Students expressed concerns about the practical aspects of incorporating AI into existing healthcare systems. This theme was moderately prevalent, indicating it's a significant consideration but not as pressing as some others. A postgraduate questioned: "How will AI tools integrate with our current EMR systems?" This reflected awareness of the technical challenges in implementing AI within existing healthcare infrastructure. An undergraduate worried: "I worry about the learning curve for older physicians in adopting AI." This highlighted concerns about generational gaps in AI adoption and the potential challenges in widespread implementation.

Enhanced pattern recognition (high frequency)

Students reported that AI tools helped them identify patterns in patient data more quickly, enhancing their diagnostic capabilities. A postgraduate student noted: "The AI system pointed out correlations in lab results that I might have missed initially." This quote illustrated how AI could augment human capabilities in data analysis and pattern recognition.

Improved diagnostic accuracy (high frequency)

Many students felt that AI-enhanced tools improved their diagnostic skills, potentially leading to better patient outcomes. An undergraduate shared: "Using the AI-based differential diagnosis tool made me consider conditions I hadn't thought of." This statement highlighted how AI could broaden the scope of diagnostic considerations, especially for less experienced clinicians.

Concerns about over-reliance (moderate frequency)

Some students worried about becoming too dependent on AI tools, potentially compromising their ability to think independently. An undergraduate expressed concern: "I fear we might lose our ability to reason independently if we rely too much on AI suggestions." This reflected the tension between leveraging AI capabilities and maintaining critical thinking skills.

Integration challenges in clinical reasoning (moderate frequency)

Students noted difficulties in incorporating AI tools into existing clinical reasoning frameworks, highlighting the need for a balanced approach. A postgraduate student observed: "It's not always clear how to balance the AI's suggestions with what we've been taught traditionally." This comment underscored the need for guidance on integrating AI insights with traditional clinical reasoning methods.

## Discussion

The present study aimed to assess the knowledge, attitudes, and practices regarding AI in medicine among undergraduate and postgraduate medical students. The findings revealed significant differences between the two groups, with postgraduate students demonstrating higher levels of AI knowledge, more positive attitudes towards AI integration in healthcare, and greater engagement with AI tools in their medical education and practice.

The knowledge assessment results indicated a substantial knowledge gap between undergraduate and postgraduate students across various dimensions of AI in medicine. The observed knowledge gap in AI between undergraduate and postgraduate medical students, while aligning with previous studies [24,25], suggests a nuanced interpretation. Rather than simply indicating a need for more education, it may reflect an existing informal learning process within the medical education system. As students progress to postgraduate levels, they are likely to gain more exposure to AI applications in clinical settings. This interpretation calls for a formalization of AI education, building upon and structuring the implicit learning already occurring. Such formalization could involve developing explicit curricula, creating standardized learning objectives, and implementing consistent assessment methods across different stages of medical education. While bridging the knowledge gap remains important, especially for undergraduates, the focus should be on creating a continuum of AI education from undergraduate years through postgraduate training and into continuing medical education. Given the limited evidence on effective AI teaching methods in medical education, there's a clear need for research into pedagogical approaches for AI integration in medical curricula. In essence, our findings highlight not just a knowledge gap, but an opportunity to formalize and enhance an adaptive process already underway in medical education [26].

Notably, both groups exhibited positive attitudes towards AI in medicine, recognizing its potential to improve patient care and enhance clinical decision-making. This finding resonates with recent studies highlighting the optimism of healthcare professionals toward AI [27,28]. However, the results also reveal concerns about job security, ethical implications, and the need to preserve the human touch in medicine. These apprehensions mirror the ongoing debates in the medical community about the disruptive potential of AI and the importance of maintaining the empathetic aspects of patient care [29,30].

The qualitative themes provide deeper insights into students' perspectives on AI in medicine. The high prevalence of the "need for AI education" theme underscores the necessity of integrating AI training into medical curricula [31]. Students' enthusiasm about AI's potential, tempered by concerns about over-reliance and integration challenges, highlights the need for a balanced approach in AI education that emphasizes the complementary role of AI in augmenting human capabilities [32].

The practice assessment results, revealing disparities in AI tool usage between undergraduate and postgraduate students, suggest that AI-related experiences are already being integrated into medical training, albeit informally. This points to an organic process of AI skill acquisition throughout medical education, with postgraduate students naturally encountering more opportunities to engage with AI tools due to increased clinical exposure. Rather than simply calling for early integration of AI experiences, these findings indicate a need to formalize and optimize the existing informal learning process. This approach involves mapping current AI exposure, identifying key points where AI concepts are implicitly taught, developing structured, evidence-based teaching methods, and creating a deliberate continuum of AI education from undergraduate through postgraduate years. Crucially, this interpretation underscores the need for research into how AI skills are currently being acquired informally, to inform the development of evidence-based teaching strategies that align with and enhance the natural progression of AI exposure in medical education. The focus should be on formalizing, standardizing, and optimizing the AI learning process that is already occurring organically throughout medical training [33,34].

Limitations

The study has several limitations that should be considered when interpreting the findings. Firstly, the cross-sectional design provided a snapshot of students' knowledge, attitudes, and practices at a single point in time. This precludes the ability to make causal inferences or track changes over time [35]. Longitudinal studies are needed to understand how students' AI-related competencies and perspectives evolve throughout their medical training and into their professional careers.

Secondly, the reliance on self-reported data may have introduced response bias, as participants might have provided socially desirable answers or overestimated their knowledge and skills [36]. Future research could employ objective measures of AI knowledge and competencies, such as performance-based assessments or simulated clinical scenarios, to obtain more accurate insights.

Thirdly, while the study included a diverse sample of undergraduate and postgraduate students, it was conducted in a single institution. This may limit the generalizability of the findings to other medical schools or healthcare contexts with different curricula, resources, or cultural attitudes toward AI [37].

Strengths

Despite these limitations, the study has several notable strengths. The mixed-methods approach provided a comprehensive understanding of students' AI-related knowledge, attitudes, and practices. The quantitative results offered measurable insights into the differences between undergraduate and postgraduate students, while the qualitative findings added depth and context to these differences [38].

The use of validated instruments for the knowledge and attitude assessments enhanced the reliability and validity of the findings [39]. The adaptation of these instruments to the specific context of AI in medicine ensured their relevance to the study's objectives.

The large sample size and stratified sampling technique contributed to the representativeness of the findings within the studied population [40]. The high response rate and the inclusion of participants from various stages of medical training further strengthened the study's internal validity.

Implications

The study's findings have important implications for medical education and practice. The identified knowledge gaps and disparities in AI tool usage underscore the urgent need for curriculum reform to integrate AI education throughout medical training [41]. This calls for the development of comprehensive AI curricula that cover technical knowledge, ethical considerations, and practical applications tailored to students' level of training [42].

The positive attitudes toward AI, coupled with concerns about its potential impact on job security and patient care, highlight the importance of fostering a balanced and informed perspective on AI among medical students [43]. This can be achieved through interdisciplinary learning experiences that engage students in critical discussions about the benefits, challenges, and ethical implications of AI in healthcare [44].

The study's insights into students' learning needs and preferences can inform the design of effective educational interventions and resources. This may include the development of AI-focused courses, workshops, and experiential learning opportunities that align with students' identified knowledge gaps and interests [45].

At a broader level, the study's findings contribute to the ongoing discourse on the future of medical education in the era of AI. They underscore the need for medical schools to adapt to the rapidly evolving landscape of healthcare technology and equip future physicians with the competencies necessary to navigate this new terrain [46]. This requires a proactive and collaborative approach that involves educators, researchers, clinicians, and policymakers working together to shape the future of AI in medical education and practice [47].

## Conclusions

This study revealed significant disparities in AI knowledge, attitudes, and practices between undergraduate and postgraduate medical students, suggesting an organic process of AI skill acquisition throughout medical training. Rather than indicating a simple need for more AI education, our findings point to the necessity of formalizing and optimizing existing informal learning processes. The challenge lies in developing a structured continuum of AI education that builds upon the natural progression of exposure from undergraduate through postgraduate years. This approach requires mapping current AI integration points, creating standardized learning objectives, and implementing evidence-based teaching methods. Future research should focus on understanding how AI skills are informally acquired and evaluating the effectiveness of various educational strategies. Crucially, as we enhance AI education, we must maintain a balance between fundamental clinical skills and ethical considerations. By addressing these challenges, medical education can evolve to prepare physicians who can effectively and responsibly leverage AI technologies in healthcare, ultimately improving patient care in the era of AI-augmented medicine.

## Appendices

**Table 8:** Questionnaire used in the study 5-point Likert scale: 1 = strongly disagree, 2 = disagree, 3 = neutral, 4 = agree, 5 = strongly agree AI: artificial intelligence; PG: postgraduate

Section	Question	Response type
1. Demographics		
	1.1 Age	Numeric input
	1.2 Gender	Multiple choice: Male / Female / Prefer not to say
	1.3 Level of study	Multiple choice: Undergraduate / Postgraduate
	1.4 Year of study	Multiple choice: 1st / 2nd / 3rd / 4th / 5th (for PG)
2. AI Knowledge Assessment		
Basic AI Concepts	2.1 What is machine learning?	Multiple choice
	2.2 Define neural networks	Multiple choice
AI Applications in Medicine	2.3 Name an AI application in radiology	Open-ended
	2.4 How can AI assist in drug discovery?	Multiple choice
Limitations of AI in Healthcare	2.5 What is a potential limitation of AI in diagnostics?	Multiple choice
	2.6 Describe a challenge in implementing AI in clinical practice	Open-ended
Ethical Considerations	2.7 What is an ethical concern in using AI for patient data analysis?	Multiple choice
	2.8 How can bias in AI algorithms affect healthcare delivery?	Open-ended
AI in Clinical Reasoning	2.9 How can AI support clinical decision-making?	Multiple choice
	2.10 Describe a scenario where AI could enhance diagnostic accuracy	Open-ended
3. Attitudes Toward AI		
	3.1 AI will improve patient care	5-point Likert scale
	3.2 AI may replace human doctors	5-point Likert scale
	3.3 AI is essential for future medical practice	5-point Likert scale
	3.4 I feel comfortable using AI in my future practice	5-point Likert scale
	3.5 AI may lead to loss of human touch in medicine	5-point Likert scale
	3.6 AI can enhance clinical reasoning skills	5-point Likert scale
	3.7 AI tools are useful in clinical reasoning education	5-point Likert scale
4. AI Practice and Experience		
	4.1 Have you used AI tools in your medical studies?	Yes/No
	4.2 Have you attended any AI in Medicine workshop/seminar?	Yes/No
	4.3 Have you read scientific papers on AI in medicine?	Yes/No
	4.4 Have you participated in AI-related research?	Yes/No
	4.5 Do you plan to integrate AI into your future practice?	Yes/No
	4.6 Have you been exposed to AI-enhanced clinical reasoning tools?	Yes/No
	4.7 Have you used AI-based clinical decision support systems?	Yes/No
	4.8 If yes to any above, briefly describe your experience	Open-ended
